# GPs’ views on the use of depression screening and GP-targeted feedback: a qualitative study

**DOI:** 10.1007/s11136-020-02703-2

**Published:** 2020-11-29

**Authors:** Lea-Elena Braunschneider, Marco Lehmann, Julia Luise Magaard, Tharanya Seeralan, Gabriella Marx, Marion Eisele, Martin Scherer, Bernd Löwe, Sebastian Kohlmann

**Affiliations:** 1 Institute and Outpatients Clinic for Psychosomatic Medicine and Psychotherapy, University Medical Center Hamburg-Eppendorf, Building West 37, Martinistr. 52, 20246 Hamburg, Germany; 2 Institute and Outpatients Clinic of Medical Psychology, University Medical Center Hamburg-Eppendorf, Hamburg, Germany; 3 Department of General Medicine, University Medical Center Hamburg-Eppendorf, Hamburg, Germany

**Keywords:** Depression, Screening, Feedback, General practitioners, Primary care

## Abstract

**Purpose:**

The first aim of this qualitative study was to identify general practitioners’ (GPs’) views on depression screening combined with GP-targeted feedback in primary care. The second aim was to determine the needs and preferences of GPs with respect to GP-targeted feedback to enhance the efficacy of depression screening.

**Methods:**

A semistructured qualitative interview was conducted with officially registered GPs in Hamburg (Germany). Interviews were audio recorded and transcribed verbatim. An inductive approach was used to code the transcripts.

**Results:**

Nine GPs (27 to 70 years; 5 male) from Hamburg, Germany, participated. Regarding depression screening combined with GP-targeted feedback, five thematic groups were identified: *application of screening; screening and patient–physician relationships; GPs’ attitudes towards screening; benefits and concerns related to screening; and GPs’ needs and preferences regarding feedback.* While the negative aspects of screening can be described in rather general terms (e.g., screening determines the mental health competence, screening threatens the doctor–patient relationship, revealing questions harm the patients), its advantages were very specific (e.g., promoting the identification of undetected cases, relief of the daily workload, wider communication channel to reach more patients). Standardized GP-targeted feedback of the screening results was perceived as helpful and purposeful. GPs preferred feedback materials that eased their clinical workload (e.g., short text with visuals, pictures, or images).

**Conclusion:**

Addressing GPs’ needs is essential when implementing depression screening tools in clinical practice. To overcome prejudices and enhance the efficacy of screening, further education for GPs on the purpose and application on depression screening may be needed. Standardized GP-targeted feedback in combination with depression screening could be the missing link to improve the detection of depression in primary care.

**Electronic supplementary material:**

The online version of this article (10.1007/s11136-020-02703-2) contains supplementary material, which is available to authorized users.

## Introduction

Major depression is one of the most disabling disorders worldwide and affects one out of every ten individuals over their lifetime in Germany [1]. International studies estimate that almost one out of every six patients in primary care suffers from depression [2]. Regarding the detection of depression, general practitioners (GPs) hold the key position in most health care systems: most are diagnosed in primary care, and most antidepressants are prescribed by GPs [2]. However, studies show that over half of the cases remain undetected [3, 4]. To close this gap, standardized depression screening in primary care could enhance the currently low detection rates. The efficacy of standardized depression screening in primary care is largely debated [5–7]. To understand how depression screening in primary care could be efficacious, the identification of the perceived benefits and concerns from GPs’ point of view is required.

In the German health care system, GPs are the first point of contact in the context of responsibility for diagnosing depression, choosing treatment and referrals. Since GPs are long-term caregivers and consultants for their patients, they play a major role in the detection of depression [8]. The German national guideline for depression, however, does not recommend standardized depression screening to support GPs’ work [7]. Despite the important role of GPs regarding patient referral and continued treatment for depression, only a few studies have investigated GPs’ views on depression screening. These qualitative studies conclude that GPs highlight more disadvantages than actual benefits. GPs doubt the validity of screening instruments [9]. Furthermore, they fear that questionnaires may threaten the patient–physician relationship [10]. On the other hand, GPs consider screening useful to verify a clinical impression, to feel more confident in detecting depression and to communicate the diagnosis [11]. To date, research has focused on the screening tool itself but has not examined how the screening result is delivered to the GP. Following a holistic screening approach, the delivery of the screening result also belongs to the screening process. However, to the best of our knowledge, no study has investigated the preferences of how screening results are delivered to the provider.

One potential way to deliver screening results is to use standardized feedback. There is evidence that standardized feedback of the screening results addressed to patients can decrease depression severity [12, 13]. Potentially, standardized feedback to GPs could also enhance the efficacy of depression screening. However, no study has investigated GPs’ views on feedback interventions in combination with depression screening. As studies have shown that health care provider-targeted feedback interventions are more effective than nontargeted interventions [13], an investigation of the needs and preferences of providers could enhance screening efficacy. Therefore, this study aims (a) to elicit GPs’ views on depression screening and depression screening with feedback and (b) to evaluate GPs’ needs and preferences regarding optimal feedback.

## Methods

### Context

This study was conducted in preparation of the multicenter randomized controlled trial GET.FEEDBACK.GP [14] (ClinicalTrials.gov identifier: NCT03988985), which tests a patient-targeted feedback intervention as an adjunct to depression screening in primary care. The design of this study and its methods applied the consolidated criteria for qualitative research (COREQ scale) [15].

### Data collection

#### Sampling

Based on the public official registry of GPs in the Hamburg region (Germany), GPs were invited to take part in the study. The only inclusion criterion was to be a registered German GP with regular patient contact. GPs received no reimbursement for participation.

#### Interview guideline and material

Following the content analysis approach [16], the aim of this study was to understand the social realities and cultural insights of GPs (as a unique cohort) while systematically sorting and comparing information to summarize them. Qualitative interviews were seen as appropriate to give every GP the possibility to explain their social reality and personal view. A semistructured interview guideline was developed (LEB, TS, JM, SK) based on an interview manual that was used in a qualitative focus group study investigating patients’ preferences and needs regarding depression screening and patient-targeted feedback [17] (see Online Appendix A). This was done to ensure similar prerequisites for the preparation of the different feedbacks in the context of the GET.FEEDBACK. GP RCT [14].

The first part of the semistructured interview included questions relating to GP’s view on depression screening combined with GP-targeted feedback. A mixture of open and broad questions was formulated to capture specific aspects of knowledge and experience with screening tools and to assess general opinions and attitudes. To illustrate a depression screening tool, GPs were shown an unfilled and completed Patient Health Questionnaire-9 (PHQ-9) [18]. The completed PHQ-9 questionnaire fulfilled the criteria of having severe depressive symptoms [18] (see Online Appendix B). GPs were invited to comment on the questionnaire and interpret it.

The second part of the interview aimed to evaluate the needs and preferences of GPs regarding optimal feedback. Six different feedback versions were provided from previous studies and media campaigns. The first version included text (54 words) stating the suspected diagnosis of depression and treatment recommendations. The text is illustrated with a red traffic light. This feedback was used in the DEPSCREEN INFO study and originally designed for cardiologists [12]. The second version included a text (73 words) stating the suspected diagnosis of depression and treatment recommendations. This feedback was provided by the German Depression Aid [19]. The third version included a text (286 words) stating the suspected diagnosis of depression and summarizing the main aspects of the national guidelines on depression treatment [20]. The fourth version displayed an illustration of a risk profile of the prevalence of depression within 100 individuals. One individual is highlighted according to the measured severity of the symptoms (13 words). This visualization was designed according to evidence-based recommendations and used in DEPSCREEN INFO [12]. The fifth version included an illustrated temperature scale combined with an illustrated risk profile (24 words) [21]. The sixth version included a text (81 words), an illustrated temperature scale, and an illustrated risk profile [22]. GPs were encouraged to discuss the different aspects of these feedback versions and to favor or disfavor specific elements of them.

#### Interview procedure

All interviews were conducted by one female PhD student (LEB, MSc Clinical Psychology) who was trained in standard interview techniques. Participants did not know LEB prior to the interviews. Interviews were conducted in line with the interview guidelines. Due to the clinical duties of GPs, some interviews were held in their practices face-to-face (*n* = 4) and some via phone (*n* = 5). For the phone calls, the GPs received all the documents via e-mail and watched them on computer screens. An interview lasted on average one hour and was audio recorded. No additional field notes were collected.

### Data analysis

The recorded interviews were transcribed verbatim by two trained external transcribers. The transcripts were checked against the audio recordings by LEB to verify their correctness. Transcripts were not returned to GPs for comments. To manage the interview data, the MAXQDA software package for qualitative analysis was used. Due to the exploratory nature of the research question, we used an inductive approach. Starting with the first transcript, LEB gradually worked through all of them. Therefore, addition coding was performed in light of preidentified codes of the previous transcripts and the new transcript material presented. These generated codes were integrated into potential thematic code groups and discussed with SK (PhD Clinical Psychology). Following this process, the consistency of codes within each thematic code group and the consistency of each thematic code group with the entire dataset were checked alternately. After repeated discussions, LEB and SK reached consensus on the thematic map and agreed on the wording of code groups and subgroups. Each quote was checked by a bilingual native German-English speaker.

## Results

### Participants

Of the 70 GPs who were approached, nine agreed to participate in the interview and completed it. No interest in the study or lack of time were the main reasons for refusal. All participating GPs gave their written informed consent, were informed about their right to stop the interview anytime, and have their interview erased from the record. The sample characteristics are shown in Table 1. Gender was balanced. Age and years of working experience showed a wide range.

**Table 1:** Characteristics of *n* = 9 general practitioners

	GP1	GP2	GP3	GP4	GP5	GP6	GP7	GP8	GP9
Gender (male)		✓			✓	✓	✓		✓
Age (years)	70	60	60	27	67	63	61	52	42
Working experience as GP (years)	30	17	35	1	32	26	29	14	2
Average patients a day	15	40	20	30	70	30	120	25	60
Estimated cases per quarter	250	900	1200	1300	2400	2000	2300	1300	1500
Estimated cases with depression per week	8	20	2	10	20	2	6	10	1
GPs who knew a depression screening tool	✓		✓	✓	✓		✓	✓	✓
GPs who use a screening tool	✓		✓				✓		
GPs who interpreted the screening result correctly			✓		✓				✓
Interview via telephone		✓			✓	✓	✓		✓

Out of nine, seven GPs knew about the PHQ-9 or similar depression screening tools. Most of them referred to their university education or further training. Three GPs used depression screening tools on a daily basis. The completed PHQ-9, indicating severe depressive symptoms, was incorrectly interpreted by six GPs who underestimated the severity of the screening result.

### GPs’ views on depression screening and depression screening combined with GP-targeted feedback

Although GPs’ views on depression screening and depression screening combined with GP-targeted feedback differed, five thematic groups emerged from the data analyses (see Table 2).

**Table 2:** Code groups and subgroups identified in the interviews

Code Groups	Subgroups	Examples
Application of screening	Unstandardized use	“It’s in my gut”
	Unclear symptoms	“Well, I do use screening when someone keeps complaining about ‘I am so tiered. I always have infections’”
	Validate presumptions	“Many patients are already familiar with this [depression screening], have googled it and have made their own diagnoses”
	Practice mental health	“It´s great for a beginner”
Screening and patient–physician relationships	Trust	“It’s not the nature of the questions [that matters], but having a relationship of trust is essential”
	Working alliance	“Not only for me, not for the file folder, but also, so I can work well with the patient”
	Objectivity	“This [feedback] is like an X-ray, a computed tomography or a laboratory examination”
GPs’ attitudes towards screening	GP’s competence	“Who else but me would know what the patient has?”
	Holistic approach	“[…] as an integral part of further differential diagnostics”
	Pressure to act	“You may end up with a mission that you did not go looking for”
Benefits and concerns related to screening	Communication	“I think it's easier for the patient to make his crosses than to tell me his complaints”
	Detection rate	“[…] this estimated number of unknown cases, to shed light on the situation”
	Effects on the patient	“I think most of them are probably feeling better after they have answered how they really feel”
	Bureaucracy	“[…] might take an enormous amount of time”
GPs’ needs and preferences regarding feedback	Clinical schedule	“Who should read all this?”
	Visualization	“[…] some signal function”
	Implementation	“If I would just get a notification on my computer and I know about it”

#### Application of screening

The ‘Application of screening’ group relates to statements concerning when screening is used. Four subgroups emerged:

*Unstandardized use* Some GPs noted that they unsystematically use depression screening: “Sometimes I do it [depression screening] intuitively or something” or “It´s in my gut.” Some GPs considered depression screening as an adjunct or rather “…as part of the anamnesis questionnaire.” One GP disagreed to use depression screening in an early stage of the medical consultation: “I consider it very difficult [to hand this out to a patient] without having a patient-physician contact beforehand.” Some GPs also stated that the patient should take the first step: “The patients must first approach me, [he/she] should have an idea that there could be something emotional behind the symptoms.”

*Unclear symptoms* Additionally, some GPs reported using depression screening, “[…] when [a patient] keeps constantly complaining about, ‘I am so tired. I always have infections.’” Screening combined with GP-targeted feedback was seen as beneficial to understand these unclear symptoms, “[…] then the whole thing gets a name. And then it becomes manageable.”

*Validate persumptions* Screening and feedback were seen as helpful to reassure a patient’s presumptions: “Many patients are already familiar with this [depression screening], have googled it and have made their own diagnoses.”

*Practice mental health* Moreover, GPs observed a practical learning effect using screening, especially for younger colleagues who just started working. “It´s great for a beginner.”

#### Screening and patient–physician relationship

The ‘Screening and patient–physician relationship’ group relates to statements concerning the possible influence of the physician–patient relationship. Three subgroups emerged:

*Trust* GPs emphasized that the efficacy of depression screening depends on the patient–physician relationship. “It´s not the nature of the questions [that matters], but having a relationship of trust is essential.” Some GPs, therefore, would not hand out a screening questionnaire to a patient without having a stable basis of trust. In contrast, some GPs stated that depression screening itself “could bridge the gap” and could be a useful tool to establish a trustful patient–physician relationship: “That´s really good from the start. Then, you have a relationship with the patient.” GPs considered that “patients feel […] seen.”

*Working alliance* Some GPs considered depression screening and GP-targeted feedback to be not only as part of the medical routine but also an opportunity for a patient–physician working alliance: “Not only for me, not for the file folder, but also, so I can work well with the patient.” In contrast, some GPs judged depression screening and GP-targeted feedback as a threat to the alliance, as one GP said: “[…] I would feel like betraying the patients.”

*Objectivity* However, GPs assumed that GP-targeted feedback could lead them to convey the diagnosis: “[…] I think […] such an objectification of the diagnosis is always important for the patient. This [feedback] is similar to an X-ray, a computed tomography or a laboratory examination. This has an effect on the patient […]. In my experience, this is convincing for the patient.”

#### GP’s attitudes towards screening

The ‘GP’s attitudes towards screening’ group relates to the statements concerning GPs’ perceived own competence and the use of screening with GP-targeted feedback. Three subgroups emerged:

*GP’s competence* One GP perceived depression screening as competing with his competence: “But I know Mr. [name]—that's my job to know him.” Another noted: “Who else but me would know what the patient has?” and that “I trust myself more than any questionnaire in the world.” GPs reasoned that they would not need a depression screening to detect depression according to their clinical experience: “[…] with the large number of my chronic patients, I can handle it without such a questionnaire.” In contrast, others emphasized the use of GP-targeted feedback, especially for colleagues “[…] with no experience in psychiatry.”

*Holistic approach* GPs noted potential advantages in disease communication when using screening “[…] if you already have prediagnosed a patient with that kind of a questionnaire before even getting to the core of the problem, then this could make communication easier.” Some GPs emphasized the usefulness of screening “as an integral part of further differential diagnostics.”

*Pressure to act* With respect to the feedback, some GPs felt the pressure to act: “You may end up with a mission that you did not go looking for.”

#### Benefits and concerns related to screening

The ‘Benefits and Concerns related to screening’ group includes statements concerning perceived benefits and concerns when using screening and feedback in their daily routine. Four subgroups emerged:

*Communication* Some GPs imagined depression screening as a time-saving tool in communication: “Sometimes I only have five minutes for a patient […]. Maybe a questionnaire like that would be helpful then.” GPs perceived a particular advantage for anxious patients: “I think it's easier for the patient to make his crosses than to tell me his complaints.” The screening tool could offer a communication channel that allows us to be open to complaints. However, GPs had concerns about whether patients with severe depression are able to fill out a depression screening: “There's someone who […] can't eat anymore or needs three days to go to the bathroom. He is afraid to speak and then you approach him with a questionnaire and he should document all this—then you will have difficulties.” Moreover, one GP worried that asking questions about depressive symptoms could be too confronting and could harm patients: “Questions do something to people. […] And they [patients] sit there on their own and are confronted.” Another GP believed, “…that they [patients] will probably end up crossing off false answers” and answer socially desired, “they would respond […] antagonistic to their inner self.” However, most GPs had no concerns that patients would fake answers. GPs noted that screening combined with GP-targeted feedback could also enhance communication. “Well, I like screening a lot, because sometimes you talk about things that suddenly pop up,” and therefore increase the quality of the consultation. “For me [as GP] as well. Sometimes it's surprising what comes out of it [the feedback].”

*Detection rate* GPs assumed that depression screening could help to identify undetected cases in primary care “[…] this estimated number of unknown cases, to shed light on the situation. This procedure could actually be helpful for that.” Moreover, they suggested that depression screening could help to structure the consultation with respect to the little time given. In addition, depression screening could monitor the course of depression and the response to treatment.

*Effect on the patient* GPs imagined benefits for the patients. Filling out a screening might help patients to cope with depressive symptoms: “I think most of them are probably feeling better after they have answered how they really feel.” Patients may be more likely to realize that they have depression: “That the patient suddenly notices, ‘Oh God, this could also be a depression.’ Some people already suspected it, but didn't believe it.” Furthermore, they appreciated obtaining insight into the patients’ subjective complaints: “How does the patient assess himself? That is also important.” However, GPs worried that GP-targeted feedback may stigmatize patients prior to their medical consultation: “Yes, I'd like to have a chat and not be presorted into such a grid system here, classified there, before anyone has even taken a look at me.”

*Bureaucracy* However, GPs in particular feared that the screening and feedback process could lead to bureaucracy and unnecessary medical documentation, which “[…] might take an enormous amount of time.”

#### GPs’ needs and preferences regarding feedback

In addition to the GPs’ general view on depression screening and depression screening combined with GP-targeted feedback, some specific formal aspects of GP-targeted feedback could be identified:

*Clinical schedule* GPs often referred to their busy clinical schedule and their need for clear and time-saving structures while commenting on the feedback material. A spontaneous reaction to text-based feedback was: “Who should read all this?” Another GP emphasized structure: “Well it´s a text, I have to read all of it. It is not grouped […] into categories of results and recommendations. It´s just […] text.” Another GP preferred “No text, actually.”

*Visualizations* Visualizations were preferred over written information because of their brevity and simplicity. Images should have “some signal function.” Thus, GPs indicated all graphic information as helpful, and no single image was generally preferred over the other. As the traffic light is a commonly used schema for GPs in Germany (e.g., for pharmaceutical budget), comments differed slightly: “That´s how GP think,” “all GPs like it that way” versus “[…] the traffic light. I immediately dislike that.” In comparison, the temperature scale was not discussed as contrarily, although it communicates the same information as the traffic light. Additionally, two GPs suggested using the risk profile as a communication tool to work with the patient. They found the table showing the prevalence icon arrays helpful, as it demonstrated that the patient is “[…] not alone. There are a lot of people who have that, too.” One GP interpreted the risk profile as a treatment aim: “[…] here, look, there's a way. That's where we want to go. Now we have to think about how to reach there?”.

*Implementation* Although the feedback should be brief to avoid wasting time, some GPs emphasized that additional guideline recommendations for depression could be helpful, especially for colleagues not primarily treating depression. Thinking about the implementation, GPs suggested including feedback to their practice software. One GP said, “If I would just get a notification on my computer and I know about it.” This would simplify medical documentation and remove potential bureaucratic concerns.

## Discussion

The first aim was to identify GPs’ views about depression screening combined with GP-targeted feedback. While negative attitudes were seen as rather general (e.g., screening determines the mental health competence, screening threatens the doctor–patient relationship, revealing questions harm the patients), the advantages of this method were very specific (e.g., rising identification of undetected cases, relief of the daily workload, and wider communication channel to reach more patients). Interestingly, screening was discussed more controversially than GP-targeted feedback. The second aim was to derive the needs and preferences of GPs regarding GP-targeted feedback. GPs prefer feedback that is visualized, well-structured, and brief to avoid wasting time during their busy working schedules.

According to our results, GPs’ views on depression screening can be qualitatively grouped into four thematic groups. Statements of the ‘Application of depression screening’ group indicate that GPs tended to use screening unsystematically. The purpose of screening was to verify their clinical impression. In line with our results, Pettersson et al. [10] showed that GPs had practical issues when introducing screening tools and integrating them in the consultation. Statement relating ‘Screening and patient–physician relationship’ indicated that some GPs felt the working alliance between the patient and physician may be threatened by screening. However, other GPs saw screening as an opener for consultation. Moreover, feedback was compared to laboratory test results, which makes the screening results objective. In line with this result, Dowrick et al. concluded that patients perceived the screening results as an objective adjunct to the medical judgment [23]. Statements in the third group, ‘GP’s attitudes towards screening,’ referred to the anticipated consequences of a standardized screening in primary care. Some GPs described the uselessness of screening, as they feel able to detect and diagnose depression without using screening tools. This view contracts with findings showing that perceived diagnostic concepts of GPs differ from those of classification systems (e.g., ICD-10) [9]. GPs tend to rely on their own experience as the gold standard [10, 24], which might be one reason for low detection rates in primary care [2]. Of note, in our sample, six out of nine GPs underestimated the severity of the screening result. Feedback after screening may help to increase the detection rate, as GPs perceive this as objective test verification. The possibility of addressing depression directly with depression screening and GP-targeted feedback was seen as a time-saving tool in the first place. The fourth group included ‘Benefits and Concerns related to screening.’ GPs appreciated that screening might help patients to express themselves, and that feedback opens a new channel for the GP to reflect patients’ symptoms. Likewise, Tavabie and Tavabie [11] found that experienced GPs benefited from screening, as it was used as a communication tool; less-experienced GPs perceived the advantages of using the screening to help with the diagnosis, which made the GPs feel more confident. However, in our study, most GPs were afraid of an increase in bureaucracy.

Next to the four groups on screening, we aimed to identify ‘GPs’ needs and preferences regarding GP-targeted feedback.’ To the best of our knowledge, this is the first study that qualitatively investigated what GPs think about feedback as an adjunct to depression screening. According to our results, GP-targeted feedback should account for the busy clinical schedule, which should be brief and illustrated with visualizations. Statements indicated that acceptance can further increase, if feedback leads to an actual reduction in workload. Since our results show that GPs can designate certain needs and preferences regarding optimal feedback, we assume that if the needs and preferences of GPs are taken into account, general barriers to the detection of depression can be overcome.

### Limitations

First, the recruitment method was a potential source of selection bias as the participants participated voluntarily without compensation. Second, the sample size was relatively small and based in the same city area, but the sample characteristics were diverse [25]. Third, some interviews were conducted via telephone, and other were conducted in person. The lack of visual communication can make a telephone conversation feel less personal and more anonymous but could enhance the interviewee’s openness. Fourth, a potential risk in interviews is that the interviewees modify their opinion to please the interviewer or to avoid conflicts. The interviewer (LEB) did not have the impression that the participants had any hesitations about expressing conflicting opinions during the interview. Fifth, regarding the analysis, potential risks can result in decontextualization of speakers’ words, which may lead to misinterpretation. However, we took care to analyze words in the broader context to ensure a faithful interpretation.

### Implications for clinical practice

The implementation of routine depression screening in primary care is much debated, primarily because efficacy trials have shown mixed results. Our results indicate that one reason for the low efficacy could be the incorrect application of screening. GPs tend to use depression screening randomly. However, depression screening is defined as a systematic application to identify individuals [26]. Additionally, GPs perceived screening as a threat to their competence. Standardized GP-targeted feedback as an adjunct to screening was perceived as an objective test result (comparable to a blood test), which was interpreted as rational why to perform depression screening. This emphasizes the importance of standardization—both for the application of screening and the feedback of the result.

In primary care studies, the term “standardized depression screening” is widely used. Often it remains unclear how “standardized” is actually defined (i.e., who receives screening, where is screening conducted, who performs the screening, calculates the scoring, communicates the results, etc.). To date, there is no clear consensus regarding what prerequisites are necessary for depression screening to be standardized. International recommendations on depression screening in primary care also do not provide a clear definition. Often, they refer to the consequences of depression screening stating that an adequate referral and treatment system should be in place [5]. However, there is no clear consensus that defines an adequate referral and treatment system for depression. To investigate how depression screening in primary care could be efficacious, we believe that it is necessary to derive quality standards for depression screening.

## Conclusion

The views of GPs on depression screening differ widely, ranging from general negative attitudes to very specific benefits. Our results provide the first insights into how GPs’ needs could be addressed when implementing depression screening tools in clinical practice. Nevertheless, to overcome prejudices and enhance the efficacy of screening, further education for GPs on the purpose and application of depression screening may be needed. Standardized GP-targeted feedback in combination with depression screening could be the missing link to improve the detection of depression in primary care.

## Electronic supplementary material

Below is the link to the electronic supplementary material.

Supplementary file1 (PDF 1043 KB)
