# Supplementary material for: GPs’ views on the use of depression screening and GP-targeted feedback: a qualitative study
Source: Qual Life Res. 2020 Nov 29;30(11):3279–86. doi: 10.1007/s11136-020-02703-2 (PMC8528735; doi:10.1007/s11136-020-02703-2)
Supplement: Supplementary file 1 — Supplementary file1 (PDF 1043 KB) [file 11136_2020_2703_MOESM1_ESM.pdf]

Braunschneider, L.-E., Lehmann, M., Magaard, J.L., *et al.* GPs' views on the use of depression screening and GP-targeted feedback: a qualitative study. *Quality of Life Research* (accepted for publication).

Appendix

A. Qualitative Interview Guide

B. Completed PHQ-9

A. Qualitative Interview Guideline

Introduction - 10 min.

- Greeting (short introductory words, thank you for participation etc.)
- Presentation of the general conditions and today's procedure
    - o We have about 45-60 minutes.
    - o With your permission, I will record this interview for study purposes...
    - o I would ask you to answer as freely as possible. In particular, I would like to hear about your assessments, experiences and opinions …
- Start audio recording

	**tasks and question**	**material / methods**
Part I: Screening – experiences and thoughts.		
1.	Introducing GET.FEEDBACK.GP	
2.	- Now that I have briefly presented the study to you, I would like to know whether you have any experiences in using instruments like this. Do you use (Depression) screening in everyday practice?<br>- Present PHQ-9	Unfilled PHQ -9
2.1.	If yes: Please tell me more about it …<br>If no: Why do you think you had not used it before / had no contact to depression screening?	
2.1.1.	If yes: Please describe how the screening had an influence on the further course of treatment (or: on your relationship with the patients). Maybe there were changes after the screening? Please tell me more. Maybe you can think of some examples.	
3.	Imagine a patient just completed the screening. Here it is. What does this form of feedback tells you?	Completed PHQ-9
4.	Can you imagine possible difficulties in using such a screening or can you tell me about experiences you remember? What experiences?	
4.1.	How could these difficulties be eliminated/reduced?	
5.	And can you imagine possible advantages in using such a screening or can you tell me about experiences you remember? What experiences?	
6.	In general, how helpful do you consider it to use such an instrument in your (practice) everyday life?	

7.	Are there any aspects that we have not yet named, but which you consider important?	

Part II: feedback		
1	Till now we were just talking about the screening, the PHQ-9. Imagine getting a feedback for the PHQ-9.<br>This could look like this one, for example.<br>What do you think of that?	feedback 1
2	Next, I would like to show you some other drafts of such feedback reports. We are interested in your opinion on this. The main focus should be on how helpful and understandable the samples are for you.	feedback 2 - 3 (text)<br><br>feedback 4 - 6 (illustration)
3	• Prioritize:<br>Which of these feedbacks do you find best? Which one maybe the worst?<br>• Which aspects of the feedback do you like? Which doesn't?<br>• Discussion:<br>a) What about this feedback is …<br>o … more / less helpful?<br>o … misleading / understandable?<br>o … which visual design do you like best? Why?<br>o Do you wish guideline recommendations?<br>b) What about general aspects?<br>o salutation,<br>o design,<br>o layout<br>o language,<br>o presentation<br>• Additions and improvements:<br>Are you missing something?	

- **End**
- **Summarizing response and appreciation to the participants**
- **Say goodbye**

B. Completed PHQ-9

Over the last two week, how often have you been bothered by any of the following problems?

	Not at all	Several days	More than half the days	Nearly every day
a. Little interest or pleasure in doing things	☐ 0	☐ 1	☒ 2	☐ 3
b. Feeling down, depressed, or hopeless	☐ 0	☐ 1	☒ 2	☐ 3
c. Trouble falling or staying asleep, or sleeping too much	☐ 0	☐ 1	☒ 2	☐ 3
d. Feeling tired or having little energy	☐ 0	☐ 1	☒ 2	☐ 3
e. Poor appetite or overeating	☐ 0	☒ 1	☐ 2	☐ 3
f. Feeling bad about yourself, or that you are a failure, or have let yourself or your family down	☐ 0	☒ 1	☐ 2	☐ 3
g. Trouble concentrating on things, such as reading the newspaper or watching television	☐ 0	☐ 1	☒ 2	☐ 3
h. Moving or speaking so slowly that other people could noticed. Or the opposite being so fidgety or restless that you have been moving around a lot more than usual.	☒ 0	☐ 1	☐ 2	☐ 3
i. Thoughts that you would be better off dead, or of hurting yourself	☐ 0	☒ 1	☐ 2	☐ 3
